# Arctic warming drives striking twenty-first century ecosystem shifts in Great Slave Lake (Subarctic Canada), North America's deepest lake

**DOI:** 10.1098/rspb.2023.1252

**Published:** 2023-09-20

**Authors:** Kathleen M. Rühland, Marlene Evans, John P. Smol

**Affiliations:** ^1^ Paleoecological Environmental Assessment and Research Lab (PEARL), Department of Biology, Queen's University, 116 Barrie St., Kingston, Ontario, Canada K7L 3N6; ^2^ Environment and Climate Change Canada, 11 Innovation Boulevard, Saskatoon, Saskatchewan, Canada S7N 3H5

**Keywords:** diatoms, palaeolimnology, large lake, climate change, lake ice, Northwest Territories

## Abstract

Great Slave Lake (GSL), one of the world's largest and deepest lakes, has undergone an aquatic ecosystem transformation in response to twenty-first-century accelerated Arctic warming that is unparalleled in at least the past two centuries. Algal remains from four high-resolution palaeolimnological records retrieved from the West Basin provide baseline limnological data that we compared with historical phycological surveys undertaken on GSL between the 1940s and 1990s. We document the rapid restructuring of algal community composition *ca* 2000 CE that is consistent with recent increases in regional air temperature and declines in ice cover and wind speed, that collectively altered habitats for aquatic biota. This new limnological regime initiated the first observation of scaled chrysophytes and favoured the rapid proliferation of small planktonic cyclotelloid diatoms which replaced the long-established dominance of large filamentous *Aulacoseira islandica* in West Basin sedimentary records. Such abrupt transformations in the primary producers of this socioecologically valuable ‘northern Great Lake’ may have widespread implications for the entire food web with unknown consequences for aquatic ecosystem functioning and fisheries, which First Nations, Métis and other northern communities depend upon, pointing to the need for new studies.

## 1. Introduction

High-latitude regions are experiencing rapid transformations because of amplified warming and increased anthropogenic activities [1], with critical repercussions for freshwater ecosystems [2]. Arctic temperatures have risen by as much as four times the global rate over the past four decades [3], with an acceleration in warming since the onset of the twenty-first century [4,5] that are in stark contrast from the Arctic of the twentieth century [6]. Since *ca* 2010 CE, average air temperatures have risen across the circum-Arctic by at least 1°C above the 1981–2010 average [7], and freshwater ecosystems are experiencing shorter periods of ice cover [8,9], with lake ice phenology in the Canadian Arctic projected to reach conditions with no modern analogues by 2080 [10]. Such rapid Arctic warming and changes in ice cover can have pronounced effects on lake ecosystem processes that exert strong controls on primary producers, triggering clear biological responses [2,11–14]. In very deep and large high-latitude lakes, warming of surface waters, changes in lake ice phenology, increases in the duration and strength of thermal stratification, and changes in convective mixing and thermal bar circulation are particularly important for regulating internal redistribution of heat and nutrients with major implications for phytoplankton production [15,16]. Indeed, remote sensing time-series data (2003–2018) show a steady and significant increase in lake-wide primary (algal) production due to climate effects in two of the world's largest freshwater lakes, Great Slave Lake (GSL) and Great Bear Lake in the Canadian Subarctic [17]. As early as the mid-1990s, increases in algal growth were reported by communities living in the GSL watershed [18], and surface scums from algal blooms were first reported in 2013 for a bay on GSL, probably triggered by climate warming [19,20]. Increasing trends in GSL primary production were also evidenced by a long-term rise in algal biomass recorded in dated sediment cores [21]. Changes in the abundance and composition of algae will have cascading and attendant effects throughout foodwebs, substantially reorganizing aquatic ecosystem functioning and community structure [2].

Diatoms (Bacillariophyceae) are siliceous algae that are well preserved in lake sediments and are proven powerful bioindicators of environmental change throughout the circum-Arctic [11,12,22,23]. Many taxa have well-defined preferences for specific aquatic habitat type, respond rapidly to aquatic ecosystem change, and are widely used to indirectly track the timing and magnitude of climate-mediated changes such as ice cover [24,25] and lake thermal characteristics [11,12,22,26]. While considerable limnological and palaeolimnological research has now been completed on small- and medium-sized Arctic lakes [27], relatively little is known about the long-term trajectories of large and deep high-latitude lakes in response to climate change. These large northern aquatic systems typically maintain extensive seasonal ice covers that can buffer the ecological effects of Arctic warming and delay the response of diatom communities [28,29]. However, with the recent acceleration of Arctic warming, even the largest and deepest northern lakes are expected to overcome this thermal inertia, as was recently observed in Lake Hazen [13,30], the largest lake in the High Arctic. Detailed limnological data on northern lakes are limited and further research and experimentation are required to understand the lake-wide environmental consequences of changes in algal composition including bottom-up effects on food webs [31]. Although much remains unclear on how ‘northern Great Lakes' are responding to accelerated warming, palaeolimnological records of primary producers at the base of the food chain may provide essential insights on the early biological effects of multiple stressors and can serve as an important precursor of impacts to higher trophic levels that rely on algae, from primary consumers to fish species [32].

### (a) Great Slave Lake

**Figure 1.**
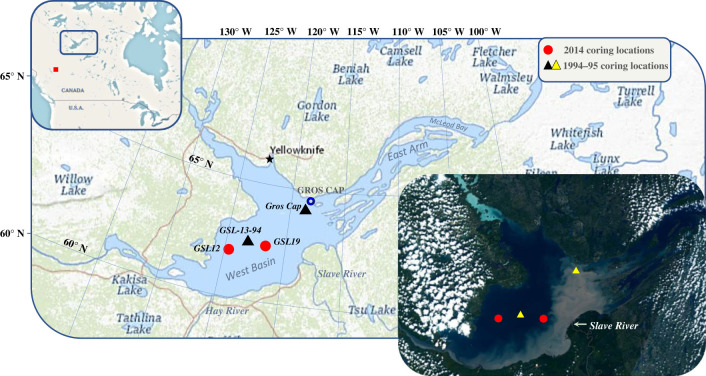
Sediment coring sites on Great Slave Lake (West Basin). Red circles depict cores retrieved in 2014 and black triangles depict archival sediment cores retrieved in the early-1990s. Upper inset: Location of GSL within Canada: the red square depicts location of the W.A.C. Bennett Dam. Lower inset: Sentinel-3-OLCI Sensor imagery taken on 2 August 2022, showing coring locations and suspended sediment discharged into the West Basin from the Slave River (Credit: European Space Agency, Sentinel Hub).

Great Slave Lake (GSL) (Northwest Territories (NWT), Canada; figure 1) is one of the world's deepest and largest freshwater lakes. Its shoreline is home to approximately 60% of the NWT population (electronic supplementary material, table S1). This large, socially and ecologically important lake has a long cultural history with First Nations and Métis communities and has supported a commercial fishery since 1945 [33]. With the exception of D.S. Rawson's intensive studies of GSL from 1944 to 1954 [34,35], few studies were undertaken during the remainder of the twentieth century and little data are available on even the baseline limnological conditions of this iconic lake. The clear dearth of scientific research on Canada's ‘northern Great Lakes' was referred to by the late David Schindler as Canada's ‘national disgrace’ [36] with a later call for renewed research efforts on these large lake ecosystems [37].

Relative to most Canadian northern lakes, Great Slave Lake has a long history of scientific surveys and limnological research, but, even so, this database pales in comparison with large temperate systems such as the Laurentian Great Lakes (LGL). Early European explorers, surveyors and scientists visited GSL including Samuel Hearne in 1771 (whose 1795 publication [38] provided information on freshwater species), Alexander Mackenzie in 1789, John Franklin in 1820, George Back from 1833 to 1835, and the first International Polar Year (IPY) expedition at Fort Rae in 1882. To date, the most comprehensive studies of GSL were initiated in 1944 by D.S. Rawson to assess the feasibility of commercial fish harvesting with expanded studies that provided highly detailed reports of the lake's physical and chemical features [34], as well as a remarkably detailed lake-wide accounting of net plankton [35] with species-level observations of phytoplankton (including diatoms). Phytoplankton sampling was undertaken during the summer (multiple times per season) from 1944 to 1947 at 57 stations across GSL, and from 1946 to 1954 at one central station (No. 31, Gros Cap). Rawson's preliminary findings of 1944 helped lead to the rapid establishment of a commercial freshwater fishery on GSL in 1945 [39].

Several decades after Rawson's study, Fee *et al*. [40] conducted a lake-wide survey of primary production in GSL during the summer of 1983 and an accounting of species composition of the dominant phytoplankton groups. Eleven years later, Evans [41] conducted a limnological study during March 1994 that focused on the West Basin and included water quality measurements and phytoplankton collection. Since 2011, Zhu *et al*. [42] have investigated zooplankton, benthos and fish populations in the GSL ecosystem. The first diatom-based palaeolimnological study of GSL [43] used a sediment core retrieved in 1987 from the very deep and clear McLeod Bay (East Arm). Other sediment studies conducted on GSL have focused on local contamination issues related to mining operations, particularly around Yellowknife Bay [44,45] and long-range atmospheric and riverine contaminant transport into GSL [46–48]. Since 2012, community-based monitoring programmes established by the GNWT (NWT-wide Community-based Water Quality Program | NWT Water Stewardship) have been collecting monthly water quality data during the ice-free season close to shore at communities such as Hay River and Fort Resolution. Despite an increase in studies, there remains a shortage of current and long-term information about the limnological conditions and algal composition of GSL. These baseline data are needed to examine the effects that human activities and climate change are having on this large and important northern aquatic ecosystem [36,49].

Here, we use dated lake sediment records to examine whether accelerated Arctic warming has affected GSL algal communities. Palaeolimnological data from a series of high-resolution ^210^Pb-dated sediment cores collected from offshore locations across the West Basin of GSL (figure 1) provide an approximately 200-year perspective of environmental change. Specifically, comparisons were made between algal analyses (diatom composition, diatom diversity and an index of scaled chrysophytes) undertaken on sediment cores collected in 2014 (GSL12 and GSL19) and on archival sediment cores (GSL13-94 and Gros Cap) that were originally retrieved in the 1990s to study contaminant trends in GSL sediments [21,47]. As the date of core collection is irrefutably the age of the undisturbed surface sediment interval, comparing algal data preserved in our new twenty-first-century sediment samples with diatom assemblages from 1990s surface sediments provides an effective means to assess whether post-2000 algal changes are exceptional over the period of the record. In addition, we use past limnological and phycological surveys of the West Basin of GSL by Rawson [35], Fee *et al*. [40] and Evans [41] to ‘ground-truth’ changes observed in our palaeolimnological records, as they provide important information on algal assemblage composition that can be compared with that same approximate period in the dated sediment records. As each sediment interval in palaeolimnological records integrates diatom taxa that have continually accumulated from different parts of the lake (space) over several growing seasons (time), these comparisons can be particularly powerful when water column samples are collected from different locations and at numerous times during the open-water season (as was done by Rawson [35], particularly at Gros Cap between 1946 and 1954) as this would incorporate a larger spatial area and a longer period of the diatom growing season. We do not make comparisons with the early McLeod Bay sedimentary diatom record by Stoermer *et al*. [43] because a reliable dating profile was not established, and, moreover, there are great differences in the physical, chemical and hydrological characteristics between the shallower, turbid West Basin (where our cores were collected) and the very deep, cold and clear East Arm where the Stoermer *et al*. [43] core was retrieved (electronic supplementary material, S1 and figure S1). Lastly, we make comparisons with regional long-term air temperature and wind speed records, and an ice phenology record that combined ground observations [50] and remote sensing data [51]. We anticipated that this very large and deep northern lake, which historical surveys indicated had maintained a relatively stable phytoplankton community dominated by the heavily silicified diatom taxon, *Aulacoseira islandica,* since at least the 1940s to the 1990s [35,40,41], may have crossed an ecological threshold and entered a new limnological regime in response to accelerated twenty-first-century regional climate change. As has been documented in numerous smaller northern lakes, longer and warmer ice-free seasons and declines in wind speed would result in changes to fundamental aquatic ecosystem processes that could restructure diatom assemblage composition [12,22]. Comparing historical limnological data, regional climate records and palaeolimnological data offers an ideal opportunity to assess the effects that rapid Arctic warming trends may have on the dynamics and structure of GSL algal communities in the context of long-term baseline conditions and can provide a critical foreshadowing of the impacts on higher trophic levels.

## 2. Site description

The Great Slave region in subarctic Canada has a northern continental, sub-humid climate with long, cold winters and short, warm summers. The Great Slave sub-basin (part of the large Mackenzie River basin) overlaps with the traditional territories of at least five First Nations and Métis groups (Akaitcho Dene First Nations, Tłı̨cho Dene First Nations, Dehcho Dene First Nations, Denesuline Dene First Nations and the Northwest Territory Métis First Nations) [49]. GSL is of great cultural and ecological importance, supporting the largest commercial, recreational and Aboriginal (CRA) freshwater fishery in the Northwest Territories and is a valuable source of Lake Whitefish (*Coregonus clupeaformis*) and Lake Trout (*Salvelinus namaycush*) for First Nations and Métis communities [42]. GSL is the second largest lake (after Great Bear Lake) that is entirely in Canada and the deepest lake in North America with a maximum depth of 614 m in the East Arm. It is a cold, slightly alkaline, oligotrophic [35,42,52], seasonally ice-covered, dimictic lake [15,53] (electronic supplementary material, table S1). Although data are limited, mean annual primary production is relatively low but has been steadily increasing, with values between 2003 and 2018 [17] estimated to be more than twice the values reported in 1983 [40], in agreement with a rise in algal biomass since the 1990s reported from GSL sediment cores [21].

GSL is bounded by two physiographic regions and three terrestrial ecozones including the Taiga Shield ecozone to the east, characterized by erosion-resistant bedrock of the Canadian Shield, and the Taiga Plains and Boreal Plains ecozones to the west differentiated by more easily erodible sedimentary bedrock of the Interior Plains [54]. The lake itself can be subdivided into various regions (figure 1) including the East Arm that includes McLeod Bay (maximum depth of 293 m) in the north, and Christie Bay (maximum depth of 614 m) in the south; waters are clear with Secchi disc depths reaching 17 m in McLeod Bay and 13 m in Christie Bay [34]. The main body of the lake is divided into the North Arm, where Yellowknife (the capital city of the NWT) is located, and the bowl-shaped West Basin (maximum depth 163 m, mean depth 41 m) which supports the GSL commercial fishery. Waters are turbid in the West Basin with Secchi disc depths ranging between 2.5 and 4.0 m [34].

One of the defining features of GSL is its very large watershed (949 000 km^2^) with the Slave River contributing an average of 71% and 99.3 km^3^ of inflow annually [55]. The river is also a major contributor of suspended sediments to GSL with estimated annual loads from 1972 to 1975 of 36.0 to 99.6 × 10^6^ metric tons yr^−1^ [44]. A turbid plume is highly visible [35] immediately after ice-off in early summer, filling much of the eastern part of the West Basin with suspended sediments (figure 1) that eventually settle and becomes diluted with less turbid GSL waters. The river sediment discharge is the main source of suspended phosphorus, nitrogen, silica and essential minerals to the lake, attributing to higher total dissolved solids and primary production in the West Basin relative to the East Arm [35,40,48] (electronic supplementary material, S2). Thus, the West Basin can support a commercial fishery while the East Arm does not. The Slave River flow has high seasonal variability, with low flows in winter and is highest in June and July; however, the construction of the W.A.C. Bennett Dam (construction and infilling completed in 1967 and 1972, respectively) altered this natural flow pattern, particularly during the infilling period [56,57]. Although difficult to measure precisely, it has been suggested that the mean annual suspended sediment load in the post-dam period at Fort Fitzgerald on the Slave River has decreased by 33%, with a 46% decrease during the ice-free period [58]. Since the Slave River sediment load greatly affects water clarity, particularly at sites located closer to the river [35,47,52] (figure 1 lower inset; electronic supplementary material, figure S3), dam construction should have resulted in an improvement in summer water clarity of the West Basin with reductions in summer flow and potentially suspended sediment load (electronic supplementary material, S3).

## 3. Material and methods

Sediment cores were collected in March 2014 using a UWITEC gravity corer by Environment and Climate Change Canada (ECCC) scientists at two sites in offshore locations of the West Basin of GSL (figure 1: GSL12—depth = 69 m: 61.37919° N, 115.35672° W; GSL19—depth = 47 m: 61.41519° N, 114.41389° W) (electronic supplementary material, S4). We also obtained two archival sediment cores that were collected 19–20 years earlier from the West Basin as part of a study investigating contaminant deposition [47] (electronic supplementary material, S4). In March 1994, site 13 (GSL-13-94: 61.40669° N, 115.00028° W; depth = 62 m) was collected by ECCC and is located between the two 2014 coring locations (GSL12 and GSL19), resulting in three West Basin sites along an east–west gradient of influence from the Slave River sediment plume (figure 1). The second archival core (GSL-12-95: 61.885° N, 113.66528° W; depth = 86 m) is located near Gros Cap (figure 1) and was collected in March 1995 by ECCC in collaboration with the Department of Fisheries and Oceans (DFO) to investigate sediment deposition at a series of sites offshore of the Slave delta where sediment deposition is more dynamic [46,47]. This most distant site was selected for analysis in this study as it is located close to Rawson's site no. 31 [35] and is hereafter referred to as ‘Gros Cap’. Archival cores do not record events after the mid-1990s.

The 2014 cores (GSL12, GSL19) were dated using a well-type gamma detector at PEARL, Queen's University, whereas dating for the two cores retrieved in the 1990s (GSL-13-94, Gros Cap) was undertaken by DFO at the Freshwater Institute (Winnipeg, Manitoba) [47] (electronic supplementary material, S5 and figure S4*a,b*). Diatom samples were processed following standard methods used at PEARL (electronic supplementary material, S6): to ensure consistency in procedures and taxonomy, diatoms from both the archival 1990s cores and the 2014 cores that were specifically collected for this study were enumerated by one scientist (K. Rühland). Principal component analysis axis one (PC1) sample scores were used to summarize the main patterns of variation in the diatom data, and non-transformed diatom relative abundance data were used to examine changes in diatom species diversity (Hill's N2 [59]). An index of chrysophyte scales to diatom frustules (S:D) was calculated for each diatom interval counted and expressed as a percentage [60].

Historical trends in climate data measured at the Hay River weather station (60.8397° N, 115.7828° W; station ID no. 2202400) provide the nearest long-term, continuous records of air temperature (1896–2015) and wind speed (1953–2015) for the West Basin of GSL (https://climate-change.canada.ca/climate-data/#/adjusted-station-data). A 54-year trend in the number of ice-free days was derived from ground observations at the Hay River station between 1956 and 1991 (station ID = WRS48) and passive microwave remote sensing data between 1992 and 2017 [51]. Ground observational ice data were accessed from the National Snow and Ice Data Center (NSIDC) [50] (https://doi.org/10.7265/N5W66HP8), and the remote sensing ice data were provided to the authors by Su *et al*. [51]. A Mann–Kendall test was applied to the climate data to determine the potential for statistically significant long-term monotonic trends in the time-series data, and Sen's slope was applied to calculate the magnitude of the trends: both methods were executed through the MAKESENS 1.0 software (https://www.researchgate.net/publication/259580998) [61].

## 4. Results

**Figure 2.**
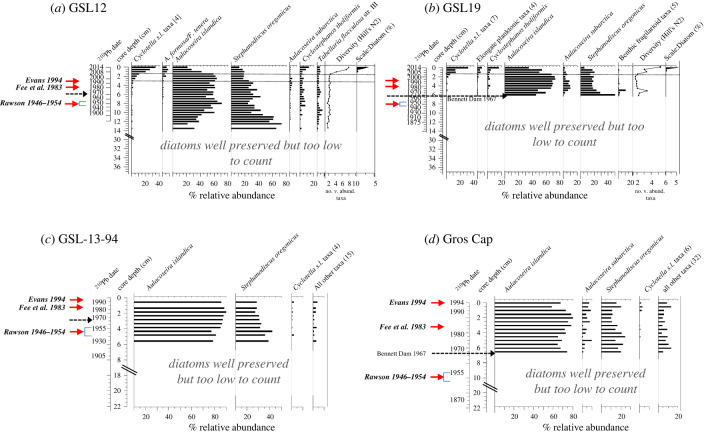
Per cent relative abundance of the most common diatom taxa identified in Great Slave Lake, West Basin sediment cores. (*a*) GSL12 and (*b*) GSL 19 are the primary cores used in this study and were retrieved in 2014. Also shown are Hill's N2 diversity trends and an index of chrysophyte scales relative to diatom frustules (Scale:Diatom). Dashed lines represent the greatest compositional change in diatom assemblages identified by clustering analysis, constrained incremental sum of squares. Archival cores (*c*) GSL-13-94 and (*d*) Gros Cap were retrieved in 1994 and 1995, respectively. Numbers in brackets following taxon names indicate the number of taxa in that grouping. Red arrows indicate the three periods of historical phytoplankton surveys in the West Basin. Black dashed arrows indicate the construction of the W.A.C. Bennett Dam.

Algal remains (diatoms, chrysophytes) were rare in the earlier parts of all four sediment records (figure 2) and enumeration was not feasible. However, the rare diatom valves encountered were in excellent condition, with no evidence of preservation issues including valve dissolution (electronic supplementary material, S3). The onset of diatom accumulation (i.e. when valves became plentiful enough to reliably establish assemblage composition) varied among the four West Basin sediment records. Diatoms became evident prior to the 1900s in core GSL12, during the 1930s in core GSL-13-94, but not until the late-1960s in cores GSL19 and Gros Cap, located closest to the Slave River plume (figure 2; electronic supplementary material, S3). Following the period of low abundance, early diatom assemblages were remarkably similar across cores and almost exclusively comprised large filamentous *Aulacoseira islandica* and large centric *Stephanodiscus oregonicus* (figure 2). Benthic taxa were rare, and only a few early intervals of the GSL19 profile recorded benthic *Fragilaria sensu lato* (*s*.*l*.) taxa (figure 2*b*). The dominance of *A. islandica* and *S. oregonicus* persisted to the mid-1990s at the tops of the GSL-13-94 and Gros Cap cores, where their sediment record ended (figure 2*c,d*) and until the late-1990s to *ca* 2000 CE in the two more recently collected GSL12 and GSL19 cores (figure 2*a,b*). Following *ca* 2000 CE, GSL12 and GSL19 registered the first observations of scaled chrysophytes, a sharp rise in diatom diversity (Hill's N2), and a pronounced shift in diatom composition (figure 2*a,b*). Post-2000 CE assemblages in cores GSL12 and GSL19 were dominated by very small-celled *Discostella pseudostelligera* (possibly *D. lacuskarluki* [62]), *Lindavia comensis*, *L. ocelleta, L. michiganiana*, and elongate planktonic *Asterionella formosa, Fragilaria tenera*, *F. delicatissima*, *Tabellaria flocculosa* str. III and *Urosolenia* taxa. By contrast, cyclotelloid and elongate planktonic diatom taxa were observed in only trace abundances in the most recent sediments of the two West Basin cores (GSL-13-94 and Gros Cap) collected in the mid-1990s (figure 2*c,d*).

**Figure 3.**
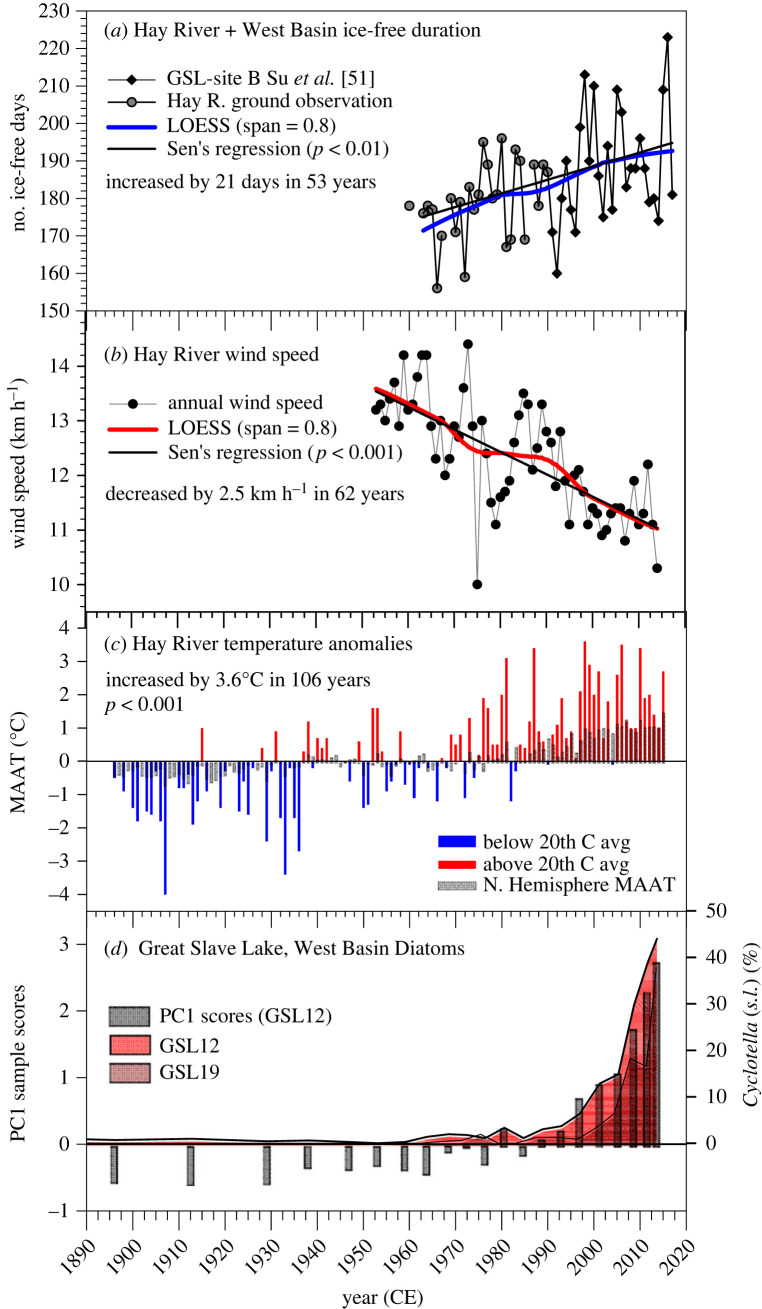
Comparison of long-term trends in sedimentary diatom data from GSL12 and GSL19 and climate trends. (*a*) 54-year composite record of number of ice-free days based on Hay River ground observations (1963–1991) and remote sensing data (1992–2017) from Su *et al*. [51]; (*b*) Hay River wind speed (1953–2015); (*c*) Hay River mean annual air temperature (MAAT) trends (1896–2015) compared with the twentieth-century average (1901–2000). The Northern Hemisphere MAAT is plotted for context; (*d*) % relative abundance *Cyclotella sensu lato* (*s.l*.) taxa from GSL12 and GSL19 and PC1 sample scores (GSL12). LOESS smoothers were used to highlight the trends in *a* and *b*, and Sen's regression was used to calculate the magnitude of the trends in *a–c*.

Mann–Kendall results determined that all climate metrics analysed (ice, air temperature, wind speed) showed a significant monotonic trend; Sen's slope calculated the magnitude of these trends (figure 3*a–c*). The number of ice-free days in the West Basin near Hay River (composite of ground observations (1963–1991) and Su *et al*. [51] remote sensing (1992–2017) data) has increased significantly (21 days in 53 years, *p*-value < 0.01) (figure 3*a*). Mean annual wind speed at the Hay River station showed a significant decrease (−0.41 km h^−1^ per decade, −2.5 km h^−1^ in 62 years, *p*-value < 0.001) from 1953 to 2015 (figure 3*b*). Hay River air temperature has increased significantly from 1896 to 2015 in all seasons including mean annual air temperature (MAAT) (0.29°C per decade, 3.6°C in 106 years, *p*-value < 0.001) (figure 3*c*) and mean winter temperature (0.47°C per decade, 5.5°C in 117 years, *p*-value < 0.001; data not shown). The Hay River MAAT data, displayed as a comparison with the twentieth-century average (1901–2000), shows cooler than average temperatures in the earlier part of the record, whereas temperatures in all years after the mid-1980s were almost exclusively warmer than average, and were particularly high after the late-1990s (figure 3*c*). The main trend in diatom assemblage composition (PC1 sample scores) in the West Basin reflects the recent shift toward dominance of small-celled cyclotelloid taxa in GSL12 and GSL19, particularly after *ca* 2000 CE (figure 3*d*).

## 5. Discussion

Great Slave Lake sedimentary records from the West Basin registered two fundamental aquatic ecosystem shifts during the past *ca* 200 years. First, with a colder climate and extensive ice covers, diatoms were rare in the earlier sedimentary sequences until algal growing conditions improved with climate amelioration in the nineteenth century (GSL12) and with accelerated warming (and flow regulation) in the twentieth century (GSL-13–94, GSL19, Gros Cap), enabling planktonic *Aulacoseira islandica* and *Stephanodiscus oregonicus* to accumulate and dominate in the sediment records (figure 2). The second regime change is a pronounced and synchronous post-2000 CE shift in diatom composition, a sharp rise in diatom diversity, and the first observations of scaled chrysophytes in cores GSL2 and GSL19 collected in 2014 (figure 2*a,b*) in response to shorter ice cover periods and reduced wind speed that accompanied accelerated twenty-first-century warming (figure 3*a–d*). Of note was the striking *ca* 2000 CE increase in small-celled, euplanktonic *Cyclotella s.l*. taxa (figure 3*d*) that rapidly displaced the long-established dominance of larger and heavier *Aulacoseira islandica* and *Stephanodiscus oregonicus* (figure 2*a,b*). Below, we discuss the diatom assemblage reorganization observed in our palaeolimnological records and use previous limnological surveys undertaken on GSL to ‘ground-truth’ the twenty-first-century shift.

### (a) Onset of diatom accumulation in West Basin sediment records

The paucity of diatoms in the early sediments of GSL12 (the longest record and located farthest from the Slave River) followed by rapid colonization of diatoms during the nineteenth century is what would be expected with the early stages of regional temperature amelioration and longer ice-free periods [27]. Similar patterns of the delayed proliferation of algal communities have been reported in diatom profiles from several deep, large, High Arctic lakes that maintain extensive ice cover even during the height of short Arctic summers [13,27] due to a strong thermal inertia [28]. In the subarctic GSL region, the onset of warmer conditions following the Little Ice Age (*ca* 1850 CE) led to reductions in water levels in the Slave River system and declines in river discharge in the Peace-Athabasca river system [63,64] that, together with longer and warmer ice-free periods, may have contributed to improved diatom growing conditions (e.g. increased water clarity) in offshore locations of the West Basin.

In comparison with GSL12, the later twentieth-century onset of diatom accumulation observed in GSL-13-94 (*ca* mid-1930s), GSL19 and Gros Cap (*ca* late-1960s) may be related to their proximity to the Slave River with its concomitant turbidity, potential dilution of diatoms with higher sediment accumulation rates (electronic supplementary material, figure S4*b*), changes in the Slave River flow regime, and the extent and distribution of the turbid sediment plume into the West Basin (figure 1; electronic supplementary material, S3). Earlier limnological surveys from the 1940s to 1990s noted that massive amounts of sediment were delivered into the West Basin following ice-off on the Slave River, resulting in highly turbid waters at offshore locations closest to the river [35,40,47,52]. Rawson [35] noted that in summers with particularly high flow, the turbid plume from the Slave River was detectable as far as Gros Cap, relatively close to our Gros Cap coring site (figure 1; electronic supplementary material, figure S3). Fee *et al*. [40] observed in their 1983 survey of the West Basin that the concentrations of the most dominant phytoplankter in GSL, *Aulacoseira islandica*, were low at stations that were directly in the turbid plume of the Slave River, suggesting that, although this taxon is tolerant of low light conditions [65], extremely turbid conditions would nevertheless negatively affect their growth. Specifically, accelerated regional warming, reduced precipitation and runoff in the Peace-Athabasca Basin [66], followed by flow regulation of the Peace River with the construction and infilling of the W.A.C. Bennett Dam, have collectively contributed to declines in the Slave River discharge [55], an increase in winter nutrients [57], changes in turbidity [42], changes in sediment and nutrient transport [67], as well as shifting the pattern of seasonal flows [56] and sediment delivery [66]. We speculate that these changes resulting from climate effects [66] and hydrological regulation starting in the late-1960s [55,58] may have altered sediment delivery patterns and increased summer water clarity at West Basin sites located closest to the river that improved algal growing conditions and facilitated diatom accumulation at our GSL19 and Gros Cap locations (figure 2*b,d*; electronic supplementary material, S3).

### (b) Early diatom assemblages of West Basin, Great Slave Lake

Early diatom assemblages were remarkably similar in all four sedimentary records from the West Basin and comprised almost exclusively planktonic taxa, dominated by *Aulacoseira islandica* and *Stephanodiscus oregonicus* (figure 2). The high representation of open-water (pelagic) taxa undoubtedly reflects the offshore locations of the cores. Although sediment cores integrate diatoms from other parts of the lake, we would expect littoral contributions (i.e. benthic taxa) to be minimal, given the relatively small littoral area of the largely pelagic West Basin. However, some spatial variability in the relative abundances of diatoms across cores would be expected (and therefore multiple cores were examined) given the large fetch, strong wave action, differences in ice phenology and turbidity across this very large lake. The prevalence of *Aulacoseira islandica* and *Stephanodiscus oregonicus* in our four GSL sedimentary records prior to *ca* 2000 CE is similar to patterns in other large, deep lakes (such as the LGL) where *A. islandica* and *Stephanodiscus* taxa are reported to co-dominate diatom assemblages during winter and then early spring, when turbulent mixing is high [65,68]. The co-dominance of these two taxa in the earlier sediments of our GSL diatom profiles can probably be attributed to a relatively short growing season and highly turbid conditions following ice-off in the West Basin as these diatoms are capable of existing in low light environments [68] and would be able to take advantage of the relatively high mineral and nutrient levels brought in from the massive Slave River sediment discharge [40,57]. Although high relative abundances of *A. islandica* and *Stephanodiscus* taxa have been associated with higher nutrient conditions in some temperate lakes [69,70], this does not appear to be the case in the early assemblages of GSL. Low plankton numbers [35], low primary production [17,40] and low total phosphorus concentrations [20,52] recorded on GSL over several decades typify oligotrophy. Rawson [35] concluded that the only strong point of agreement between the plankton of GSL and the characteristic oligotrophic plankton type based on European standards, was the great scarcity of cyanobacteria. Stoermer *et al*. [43] noted that that the co-occurrence of highly oligotrophic diatoms together with quintessential eutrophic indicators in the East Arm of GSL suggested that, in this particular case, the relatively strong presence of *Stephanodiscus* taxa was not an indication of advanced eutrophic conditions. Earlier phytoplankton surveys of the West Basin water column [35,40] did not report notable abundances of *Stephanodiscus oregonicus* that dominated our earlier sedimentary assemblages. However, this taxon was recorded in living material taken from the West Basin in the 1980s [71]. It is plausible that this taxon was present during these earlier phycological surveys, but, unlike sediment records that integrate diatom taxa over entire growing seasons (including short, intense bloom periods) and over the entire water column, a single spot sample may have missed the timing or depth where this taxon would be most prolific (electronic supplementary material, S7).

The high relative abundances of filamentous *A. islandica* in the earlier sediments of our four diatom records also agree with observations made in early phytoplankton surveys of the West Basin from the 1940s to the mid-1990s where this taxon was consistently reported to be the dominant phytoplankter throughout the West Basin [35,40,41]. For example, *A. islandica* was by far the dominant taxon of the approximately 160 algal species observed throughout GSL by Rawson in the 1940s to 1950s [35]. Using phytoplankton data collected by Rawson from 1946 to 1954 at Gros Cap (which Rawson deemed representative of the West Basin), Lund [72] observed large increases in *A. islandica* immediately following ice-out and suggested that this taxon begins to grow during ice cover and/or grows very rapidly at low temperatures. In agreement with observations by Lund [72] on GSL, this large filamentous taxon has been widely reported to bloom under ice during winter circulation in large, cold lakes, once light levels are sufficient and spring silica levels are high [69,70,73]. The high turbulence, high silica and mineral content following ice-off in the West Basin are conditions that *A. islandica* is particularly well suited for, as it can use light efficiently and thereby withstand high levels of turbidity [65]. Decades following Rawson's study, Fee *et al*. [40] similarly reported that *A. islandica* made up 78% of phytoplankton sampled immediately after ice-out in June 1983, dominating samples from stations throughout the West Basin. Evans [41] likewise noted that the March 1994 phytoplankton community in the West Basin was dominated by *A. islandica*. Our dated sediment archives provide a longer-term perspective, demonstrating that this taxon has dominated diatom assemblages from the West Basin for at least the past *ca* 200 years (until the mid-1990s).

### (c) Pronounced twenty-first-century reorganization of algal assemblages in the West Basin of Great Slave Lake

The post-2000 CE shift in dominance from the heavily silicified *Aulacoseira islandica* to more buoyant *Cyclotella s*.*l*. taxa and elongate planktonic diatoms (e.g. *Asterionella formosa*, *Fragilaria tenera*) observed in our GSL sediment records (GSL12, GSL19) is a classic diatom response to warming with longer open-water periods and reduced wind speed, that is well documented in hundreds of lake sediment records worldwide (e.g. [11,12,22,74–77]). Climate has significant indirect effects on algal species composition with the development of new habitats and by changing some of the fundamental aquatic ecosystem processes [11,12]. In particular, phenological changes in ice cover, vertical mixing and thermal stratification processes and associated changes in resource availability (light and nutrients) exert key controls on algal growth and population dynamics [12,14,78]. In conjunction with a pronounced shift in diatom composition, scaled chrysophytes occurred for the first time in countable numbers *ca* 2010 and increased thereafter (figure 2*a,b*), as might be expected with climate-related water column changes. These flagellated golden algae have low light:nutrient requirements and tend to bloom at or below the thermocline [79], and with stronger and longer periods of thermal stratification, they can benefit from nutrient-rich waters in a deeper thermocline [80].

The pronounced twenty-first-century algal shifts registered in our two 2014 GSL sediment cores agree with recent climate trends recorded at the Hay River weather station, including the highest number of ice-free days since 1963 (figure 3*a*), the lowest wind speeds since 1954 (figure 3*b*) and the highest air temperatures since 1896 (figure 3*c*). These recent climate trends for the GSL region provide ideal conditions for changes in lake physical properties that would favour a shift from heavily silicified diatoms to smaller-celled taxa [12,22,31,78]. *Cyclotella s.l*. taxa and elongate planktonic taxa that became prominent in the post-*ca* 2000 CE GSL sediments, have high surface area to volume ratios, efficient nutrient uptake and light harvesting mechanisms [81], relatively low sinking velocities [80] and are capable of prolific reproduction [82]. With a warmer and more stable water column, these ecophysiological traits are favoured [12], enabling small-celled and more buoyant diatoms to outcompete heavier, large-celled, fast-sinking diatoms such as *A. islandica* that require turbulence for resuspension of nutrients and for maintaining a desirable position in the photic zone [70]. Indeed, Lund [72] examined the periodicity of *A. islandica* using Rawson's samples collected from 1946 to 1954 at Gros Cap and described what he referred to as a striking relationship between *A. islandica* populations in GSL and vertical gradients in water column density: once turbulent mixing was reduced in summer, the *A. islandica* population sank rapidly into the hypolimnion and then to the bottom sediments.

The small-celled *Cyclotella s*.*l*. taxa, which were in high abundances after *ca* 2000 CE in our 2014 sedimentary records, were not reported in earlier phytoplankton surveys [35,40,41], nor were they registered in notable abundances in either of our 1990 archival sediment cores. It is plausible that Rawson's 1946–1954 survey of GSL phytoplankton using a #20 silk net (approx. 76 µm mesh size) would not have captured very small-celled cyclotelloid taxa that dominate our most recent sedimentary intervals (electronic supplementary material, S8). However, these small cyclotelloid taxa were also absent in the surveys by Fee *et al*. [40] and Evans [41] that captured algal biomass in the 2–40 µm range, suggesting that these small centric diatoms were not prolific in the West Basin of GSL at that time. Perhaps the strongest evidence that small cyclotelloid taxa were not yet an important component of the phytoplankton of the West Basin prior to the mid-1990s is provided by our archival 1994 and 1995 cores, where *Cyclotella s*.*l*. taxa were present in only trace abundances (less than 2%) in the surface sediment intervals that include diatoms deposited at the time of core collection. Past limnological surveys together with our archival sediment records indicate that the shift in algal composition that we report is irrefutably unique to the twenty-first century.

Another recent indication that GSL primary producers are responding to accelerated twenty-first-century warming and longer open-water periods is the first reported observations of bloom-forming cyanobacteria surface scums in Yellowknife Bay (September 2013, September 2014, August 2015) [20], during a period when the water column was stratified [19], and nutrients and productivity measurements were relatively low [20]. Colonial bloom-forming planktonic cyanobacteria have been generally absent from high-latitude freshwater systems [35], as cold temperatures would inhibit the growth of these temperature-sensitive taxa [83]. For blooms to develop on GSL would have required an ideal combination of sufficient nutrients, low turbulent mixing during stratification and sufficiently high temperatures [19]. These are the same conditions that would favour the proliferation of small-celled cyclotelloid taxa that we observed during the past few decades in our West Basin sediment records.

The restructuring of Great Slave Lake primary producers registered in our cores is a clear sign that this iconic ‘northern Great Lake’ has only recently entered a new limnological regime in response to accelerated twenty-first-century warming, declining ice covers and wind speed, and fundamental changes to lake thermal structure. Clearly, algal communities of large, deep, high-latitude lakes with extensive ice covers are no longer buffered from the ecological impacts of Arctic amplification, similar to the changes observed in many small- and medium-sized Arctic lakes since the mid-nineteenth century [11,12,22,27]. Primary producers such as diatoms often respond to environmental issues well in advance of organisms from higher trophic levels, from zooplankton that depend on algae for food, to fish species [84]. Therefore, shifts in sedimentary algae registered in palaeolimnological records provide important insights into trajectories of whole-lake ecosystem functioning [32]. Under the new climate regime of the twenty-first century, the cascading effects that a shift from large-celled to small-celled diatoms will have on higher trophic levels are unknown for GSL and would require intensive long-term limnological monitoring and studies on food preferences of grazing zooplankton. However, we speculate that a reduction in large-celled diatoms or an overall decline in diatom contributions to the phytoplankton of GSL may result in a loss of high-quality food (diatoms) for macrofauna (e.g. amphipod and mysid populations), with significant consequences to the lake whitefish population [85,86]. Similar to our findings for GSL, the diatom communities of all of the LGL have recently undergone a compositional shift towards higher abundances of small cyclotelloid taxa in response to accelerated warming [31,32]: even with years of intensive limnological research, the bottom-up effects of this diatom shift in the LGL are also currently unknown [32]. With a large portion of the NWT population living near its shorelines, GSL supports a wealth of important socio-economic services including the largest CRA freshwater fishery in the NWT [42] and, as one of the world's largest freshwater lakes, it is also an important part of the global carbon cycle [17]. Accelerated Arctic warming is rapidly pushing high-latitude freshwater ecosystems toward more temperate conditions, with unknown ramifications for fisheries and aquatic ecosystem functioning that First Nations, Métis, and other northern communities rely on.

## Data Availability

The datasets supporting this article have been uploaded to Dryad Digital Repository: https://doi.org/10.5061/dryad.5hqbzkhbs [87]. Supplementary material is available online [88].
